# Multi-omics analysis of SIV-specific CD8^+^ T cells in multiple anatomical sites

**DOI:** 10.1371/journal.ppat.1012545

**Published:** 2024-09-09

**Authors:** Jennifer Simpson, Brittany Dulek, Paul Schaughency, Jason M. Brenchley

**Affiliations:** 1 Barrier Immunity Section, Laboratory of Viral Diseases, National Institute of Allergy and Infectious Diseases, National Institutes of Health, Bethesda, Maryland, United States of America; 2 Integrated Data Sciences Section, Research Technologies Branch, National Institute of Allergy and Infectious Diseases, National Institutes of Health, Bethesda, Maryland, United States of America; National Cancer Institute, UNITED STATES OF AMERICA

## Abstract

CD8^+^ T cells exert immunological pressure against immunodeficiency lentiviruses. In previous studies, we examined the TCR repertoire of CD8^+^ T cells specific for a single SIV immunodominant epitope, Gag-CM9, throughout SIV infection or after vaccination, and across multiple anatomic sites. We identified both tissue specific TCR sequences and TCRs shared by multiple anatomical sites. Here we use single cell RNA sequencing to evaluate if the tissue localization or TCR sequence of a CM9-specific CD8^+^ T cell corresponds with unique transcriptomics. CM9-specific CD8^+^ T cells were sorted from blood, lymph nodes, spleen, and liver from SIV infected rhesus macaques with progressive SIV infection and in animals who spontaneously control SIV replication after cessation of antiretroviral therapy. The cells were processed through a single cell sequencing protocol, creating a TCR amplified library and an RNA gene expression library corresponding to individual cells. Gene set enrichment analysis revealed no distinct transcriptional profiles for CM9 specific CD8^+^ T cells between different anatomical sites and between cells with shared or tissue specific TCRs. Similarly, no clear transcriptional profiles were associated with clonotypes which were shared across individual animals. However, CM9 specific CD8^+^ T cells from posttreatment controllers did exhibit enrichment of pathways associated with cellular activation compared to progressively infected animals, suggesting that altered transcription in distinct cellular pathways in antigen specific CD8^+^ T cells may associate with viral control. Together, these studies represent a thorough analysis of the relationship between anatomical and clonal origin, and the transcriptional profile of antigen specific CD8^+^ T cells and unravel pathways that may be important for CD8^+^ T cell mediated control of SIV replication.

## Author summary

Virus-specific CD8^+^ T cells against human immunodeficiency virus (HIV), or, in nonhuman primate models, simian immunodeficiency virus (SIV), are thought to contribute to the antiviral response in acute infection. Virus-specific CD8^+^ T cells are observed in blood and multiple tissues, with some CD8^+^ T cell clones observed in a single anatomical site. Currently, it is unknown whether the anatomical site or tissue specificity of a CD8^+^ T cell clone contributes to the transcriptional phenotype of the cell. Here, we used a single-cell multi-omics approach to evaluate the transcriptional phenotype of virus-specific CD8^+^ T cells in blood and multiple tissues. We observed no association between the anatomical site and the transcriptional phenotype of the virus specific CD8^+^ T cells and observed differences in only a couple of cellular pathways between cells found in multiple anatomical sites and those found in just one site. We did identify specific pathways with differential expression between animals able to control viral infection and those with progressive infection. This study represents an in-depth analysis of the clonal origin, anatomical location, and transcriptional phenotype of virus specific CD8^+^ T cells and contributes to the understanding of the CD8^+^ T cell response in chronic viral infection.

## Introduction

After infection with human immunodeficiency virus (HIV) or, in nonhuman primates, simian immunodeficiency virus (SIV), CD8^+^ T cells are stimulated from the naïve pool and subsequently develop effector functions that contribute to the antiviral response [1–3] including cytotoxic functions [4] and release of cytokines and chemokines [4–7]. CD8^+^ T cells recognize viral epitopes presented by major histocompatibility complex (MHC) class I molecules of infected cells via the T cell receptor (TCR) of the CD8^+^ T cell [8]. The classical TCR is a heterodimer of an α and β chain. The α chain contains a variable (V) and joining (J) gene segments and the β chain contains V, diversity (D) and J gene segments. Multiple copies of these segments are encoded in the genome and somatic recombination of the V–J (TCRα) or V–D–J (TCRβ) segments generates diversity among T cells. Further diversity is generated by random nucleotide addition/deletion at the junctions between gene segments [8,9]. The junctional region between the V and J or D and J genes of the TCRβ chain is the hypervariable complementarity-determining region-3 (CDR3) and interacts most closely with the peptide/MHC complex. Therefore, the sequence of the CDR3 region is typically used to determine the TCR clonotypic profile of T cell subsets [8–11].

Previously, we studied the TCR repertoire of CD8^+^ T cells specific for a single SIV immunodominant epitope, Gag-CM9 (CTPYDINQM) during acute infection and chronic infection, after vaccine-induced T cell immunity, and in the presence and absence of antiretrovirals (ARVs). Furthermore, we studied the clonal hierarchy of the CM9-specific CD8^+^ T cells across multiple anatomic sites of the same animals. We identified some TCR clonotypes found in only one anatomical site throughout SIV infection, suggesting some CD8^+^ T cell clonotypes do not circulate throughout the body [12,13]. This is in line with extensive parabiosis studies in mice that have previously characterized non-migratory, tissue resident CD8^+^ T cells in multiple disease states [14–17]. Furthermore, previous studies have identified upregulation of tissue residency markers such CD69 and CD103 on HIV specific CD8^+^ T cells in distinct anatomical sites [18–20], suggesting establishment of tissue residency and a lack of constant recirculation of antigen specific CD8^+^ T cells. As yet, these analyses have not been replicated in SIV infected nonhuman primates.

In this study, we sought to use single cell RNA-seq to explore the transcriptomics of SIV-specific CD8^+^ T cells, and cells specific for another virus that induces chronic infection in the host, cytomegalovirus (CMV). We examined antigen-specific cells across multiple anatomic sites, coupled with TCR sequence analysis to determine if unique transcriptional profiles would differentiate tissue-specific clonotypes relative to clonotypes present simultaneously within multiple anatomic sites. We also assessed if the anatomical location of a SIV-specific CD8^+^ T cell influenced the gene expression profile. Furthermore, we compared the TCR repertoire and gene expression profiles of SIV-specific CD8^+^ T cells from animals with progressive SIV infection and those with post-treatment control of SIV. Through single cell multi-omics, we identified no association between the TCR sequence and transcriptional profile of CM9-specific CD8^+^ T cells and no substantial differences between the CM9-specific CD8^+^ T cells from multiple anatomical sites. We also observed global differences in transcriptional profile, and distinct TCR repertoires between SIV and CMV-specific CD8^+^ T cells. Additionally, several pathways were enriched in CM9-specific CD8^+^ T cells from post-treatment controllers compared to non-controllers, suggesting specific transcriptional profiles in antigen specific CD8^+^ T cells may associate with viral control after treatment cessation.

## Results

### GSEA of CM9-specific CD8^+^ T cells shows a lack of distinct transcriptional profiles between anatomical sites, with minimal clustering of RNA-seq data by animal or anatomical site

**Fig 1:**
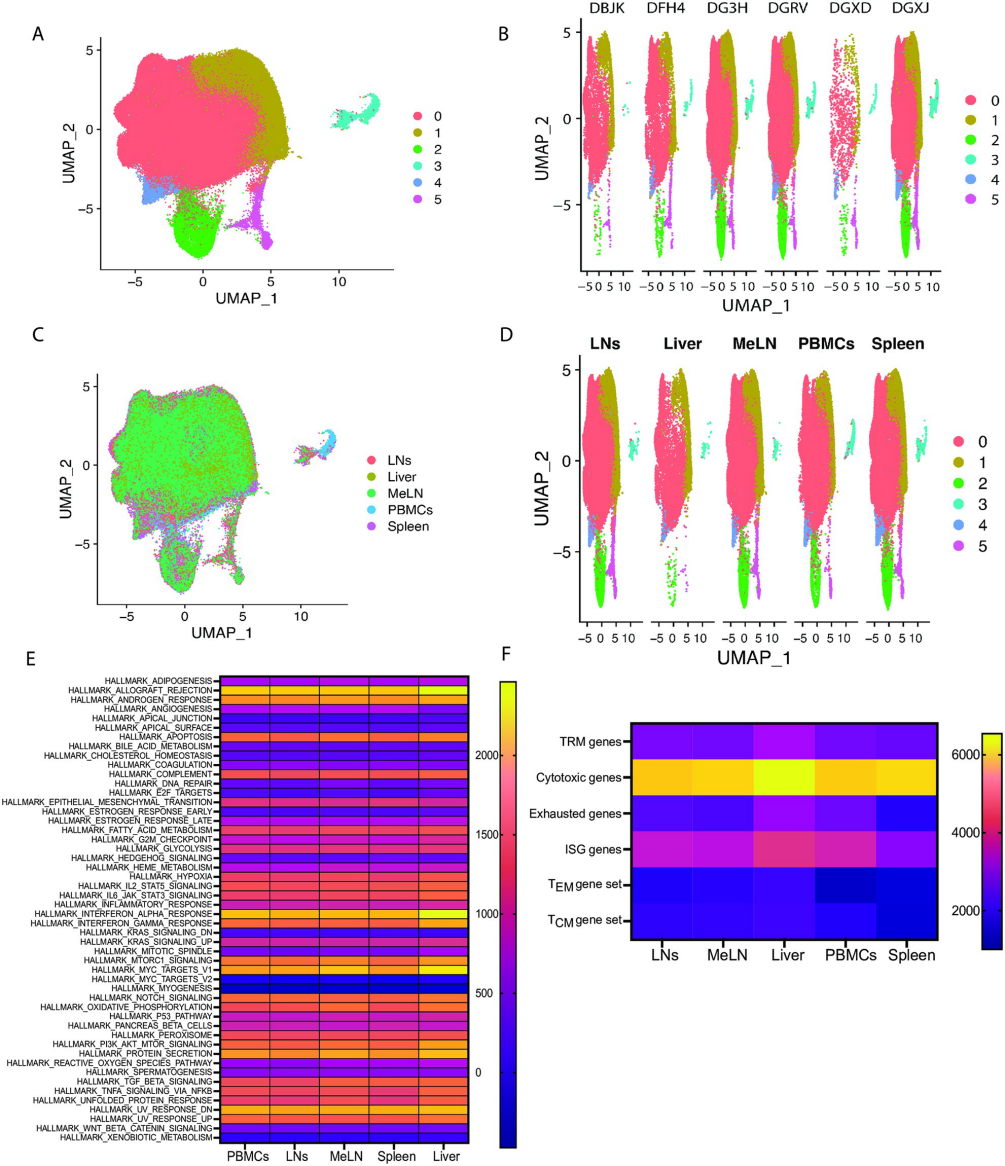
GSEA shows similar transcriptional profile between anatomical sites. (A) UMAP analysis of RNA-seq data from CM9-specific CD8^+^ T cells from multiple anatomical sites from chronically SIV infected rhesus macaques, colors designate unbiased clusters. (B) UMAP analysis of RNA-seq data from CM9-specific CD8^+^ T cells, separated by animal ID (DBJK, DFH4, DGRV, DGXD, DGXJ, DG3H) and colors designate unbiased clusters. (C) UMAP analysis of RNA-seq data from CM9-specific CD8^+^ T cells with colors designating anatomical site. (D) UMAP analysis of RNA-seq data from CM9-specific CD8^+^ T cells, separated by anatomical site and colors designate unbiased clusters. (E) Heatmap analysis of the median expression enrichment value for each of the Hallmark gene sets for each anatomical site. (F) Heatmap analysis of the median expression enrichment value for the TRM signature gene set (*ITGAE*, *ITGA1*, *RUNX3*, *NR4A1*, *CD69*), the cytotoxic signature gene set (*GZMB*, *GZMA*, *NKG7*, *FCRL6*, *SLAMF7*, *CX3CR1*, *PRF1*), the exhausted signature gene set (*PDCD1*, *LAG3*, *TIGIT*, *CTLA4*, *HAVCR2*), the ISG signature gene set (*IRF7*, *IRF2*, *IRF1*, *MX1*, *BST2*), the T_CM_ gene set (*SELL*, *CCR7*, *TBR1*, *TCF7*, *BCL6*, *ID3*, *STAT3*) and the T_EM_ gene set (*TBX21*, *ID2*, *STAT4*, *KLRG1*). Data are representative of 6 animals. Data are represented as UMAP (A—D) or heatmap (E, F) approximation. Statistical comparisons (E-F) were conducted by two-way ANOVA with Tukey’s or Sidak’s multiple comparisons test. Significance is defined as p < 0.05.

CM9-specific CD8 T cells were isolated from peripheral blood mononuclear cells (PBMCs), several non-gut draining lymph nodes including axillary, inguinal, submandibular, and cervical lymph nodes (LNs), in addition to mesenteric lymph nodes (MeLN), spleen and liver from 6 chronically SIV-infected rhesus macaques (animal information in S1 Table). CM9-specific CD8 T cells from PBMCs, LNs, MeLN, spleen and liver were then processed through single cell RNA-seq and TCR-seq pipelines. Transcriptional analysis revealed 6 distinct clusters after unbiased clustering via the R package *Seurat* (Fig 1A). To identify the cells that constituted the distinct clusters, differentially expressed gene analysis (using *Seurat’s* “Findmarkers” with Wilcox test) was conducted on each of the clusters and genes uniquely upregulated in each cluster were identified in a heatmap (S1A Fig). As the majority of cells were located in clusters 0 and 1 (S1B and S1C Fig), further analysis identified differentially expressed genes between these two clusters. This included upregulation of the CCL4 homolog *CCL4L1* [21] and the tubulin encoding gene *TUBB* [22] in cluster 0, with both genes showing downregulation in cluster 1 (S1D Fig). Elevated release of CCL4 by CD8^+^ T cells is associated with HIV viral suppression [23,24], while higher expression of *TUBB* and other microtubule-associated genes has been observed in effector memory T cells (T_EM_) derived from the tissues [22], although we saw no association between anatomical site and the unbiased clustering (S1B Fig). The 6 clusters identified by unbiased clustering were not associated with the different animals (Fig 1B). Given the use of different library preparation kit versions and sequencing machines, we were concerned there would be batch effects, however, with no obvious clustering by animal, and given different animals were processed with different kits, this suggests that batch effects due to the library preparation kit or sequencing machine are minimal. Clustering was also not associated the distinct anatomical sites (Fig 1C and 1D and S1B Fig), as all clusters were present in each animal and anatomical site.

To characterize the transcriptional profile of CM9-specific CD8^+^ T cells across the different anatomical sites, we completed GSEA, firstly using the Hallmark pathway gene sets [25]. Comparing the median enrichment score for each anatomical site for each pathway, no significant differences were observed between the anatomical sites (Fig 1E). To further characterize the transcriptional state of CM9-specific CD8^+^ T cells, unique gene sets were generated, including a tissue resident memory T cell (TRM) signature gene set (*ITGAE*, *ITGA1*, *RUNX3*, *NR4A1*, *CD69*), a cytotoxic signature gene set (*GZMB*, *GZMA*, *NKG7*, *FCRL6*, *SLAMF7*, *CX3CR1*, *PRF1*), an exhausted signature gene set (*PDCD1*, *LAG3*, *TIGIT*, *CTLA4*, *HAVCR2*), an ISG signature gene set (*IRF7*, *IRF2*, *IRF1*, *MX1*, *BST2*), a T_CM_ gene set (*SELL*, *CCR7*, *TBR1*, *TCF7*, *BCL6*, *ID3*, *STAT3*) and a T_EM_ gene set (*TBX21*, *ID2*, *STAT4*, *KLRG1*). CM9 specific CD8^+^ T cells in all anatomical sites showed a trend for low expression for the TRM signature, but a high expression of the cytotoxic signature genes, a somewhat surprising observation as SIV specific CD8^+^ T cells typically exhibit exhausted phenotypes in chronic SIV infection [26]. Comparing the median enrichment score across each anatomical site, no significant differences were observed between anatomical sites (Fig 1F), suggesting similar transcriptional profiles for CM9-specific CD8^+^ T cells regardless of their anatomical origin.

### The TCR repertoires of CM9 specific CD8^+^ T cells are similar between anatomical sites and there is no clear interaction between TCR sequence and transcriptional profile

**Fig 2:**
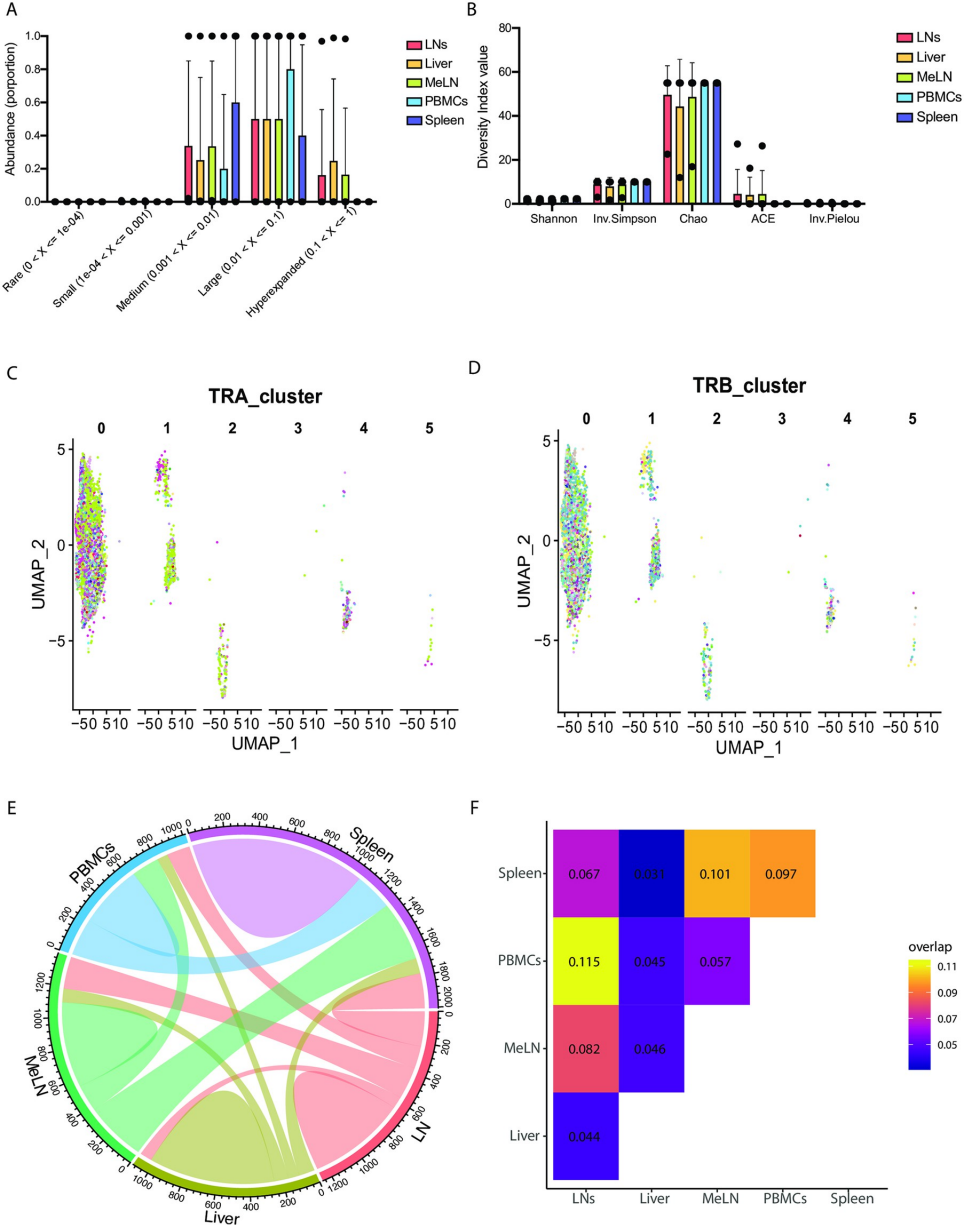
The TCR repertoires of CM9-specific CD8^+^ T cells are similar across anatomical sites and show minimal interaction between TCR sequence and transcriptional profile. (A) Relative abundance of TCR sequences from CM9-specific CD8^+^ T cells from multiple anatomical sites from chronically SIV-infected rhesus macaques. (B) Diversity index values of TCR sequences from CM9-specific CD8^+^ T cells from multiple anatomical sites. (C) TCRα chain cluster of TCR sequences from CM9 -specific CD8^+^ T cells from multiple anatomical sites, with color designating different TCRα chain clusters. (D) TCRβ chain cluster of TCR sequences from CM9-specific CD8^+^ T cells from multiple anatomical sites, with color designating different TCRβ chain clusters. (E) *Circos* plot of clonotype sharing between multiple anatomical sites. (F) Overlap plots showing the TCR repertoire overlap of CM9-specific CD8^+^ T cells from multiple anatomical sites. Data are representative of 6 animals. Statistical comparisons (A, B) were conducted by mixed-effects analysis (A), or two-way ANOVA (B) and Tukey’s multiple comparisons test. Data are represented as mean (bar) and individual values (black dots) (A, B), and UMAP or heatmap approximations (C-F).

The TCRα and TCRβ sequences from CM9-specific CD8^+^ T cells were identified for each anatomical site via single-cell TCR-seq. There were no significant differences between the anatomical sites regarding the TCR repertoire constitution, with all anatomical sites exhibiting largely oligoclonal repertoires (Fig 2A). The TCR repertoires of the CM9-specific CD8^+^ T cells from all anatomical sites were also similar regarding diversity, as determined by 5 diversity indices (Fig 2B), suggesting similar TCR repertoires between cells specific for a single epitope in different anatomical sites.

A recent report has identified a strong association between TCR sequence and transcriptional phenotype of antigen specific CD8^+^ T cells [27]. To determine if a similar phenotype was observed in our dataset, clustering was performed on the TCRα (TRA) and TCRβ (TRB) chain and applied to the RNA-seq dataset. While over 900 TCR clusters were identified, we did not see a clear association between the TCRα or TCRβ sequence and the 6 distinct clusters identified in the RNA-seq dataset (Fig 2C and 2D), although the large number of TCR clusters may have clouded this analysis.

We next sought to evaluate the sharing of the TCR sequences among anatomical sites. *Circos* plots revealed every anatomical site had a least some TCR sequences that were observed in other sites (Fig 2E). To further assess the sharing of TCR sequences between anatomical sites, overlap analysis was utilized. The TCR repertoire of CM9-specific CD8^+^ T cells from the LNs and the PBMCs showed the most overlap, with the liver having the most unique TCR sequences (Fig 2F), suggesting strong sharing of TCR sequences between lymphoid tissues, but less sharing between lymphoid and nonlymphoid tissue, consistent with our previous results [13].

### CM9 specific CD8^+^ T cells exhibit similar transcriptional profiles associated with shared and tissue specific TCR sequences

**Fig 3:**
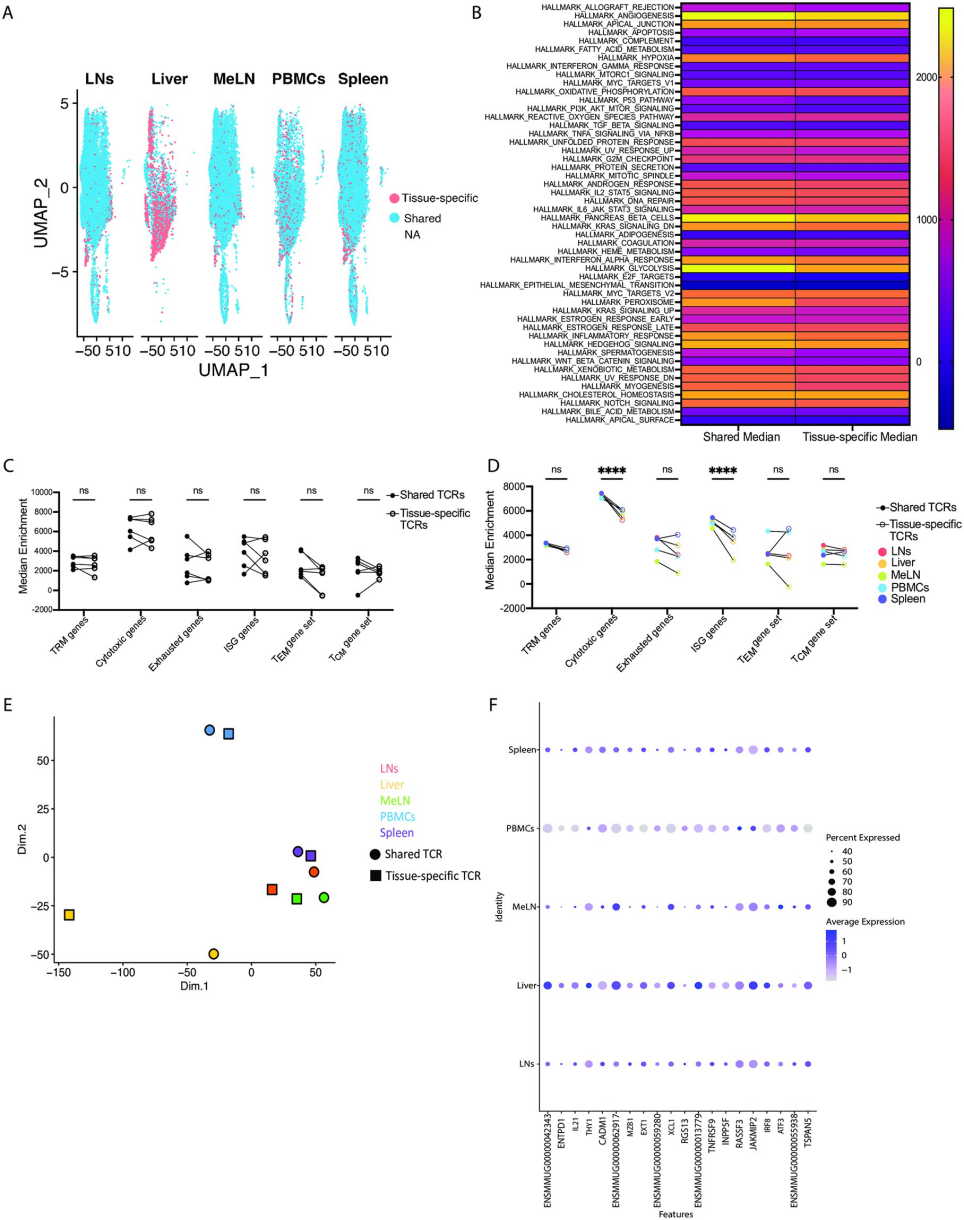
CM9-specific CD8^+^ T cells exhibit similar transcriptional profiles associated with shared and tissue specific TCR sequences. (A) UMAP analysis of RNA-seq data from CM9-specific CD8^+^ T cells from multiple anatomical sites from chronically SIV infected rhesus macaques, colors designate cells with shared or tissue specific TCRs. (B) Heatmap analysis of the median expression enrichment value for each of the Hallmark gene sets for cells with either shared or tissue specific TCRs. (C) Median gene expression levels for the TRM signature gene set (*ITGAE*, *ITGA1*, *RUNX3*, *NR4A1*, *CD69*), the cytotoxic signature gene set (*GZMB*, *GZMA*, *NKG7*, *FCRL6*, *SLAMF7*, *CX3CR1*, *PRF1*), the exhausted signature gene set (*PDCD1*, *LAG3*, *TIGIT*, *CTLA4*, *HAVCR2*), the ISG signature gene set (*IRF7*, *IRF2*, *IRF1*, *MX1*, *BST2*) the T_CM_ gene set (*SELL*, *CCR7*, *TBR1*, *TCF7*, *BCL6*, *ID3*, *STAT3*) and the T_EM_ gene set (*TBX21*, *ID2*, *STAT4*, *KLRG1*), with data separated by animal. (D) Median gene expression levels for the TRM signature gene set, the cytotoxic signature gene set, the exhausted signature gene set, the ISG signature gene set, the T_CM_ gene set and the T_EM_ gene set, with data separated by anatomical site. (E) MDS analysis of the average gene expression of all genes in cells with either shared or tissue specific TCRs for each anatomical site. (F) Dot plot representing the expression levels of the 20 most differentially expressed genes in cells from the blood. Data are representative of 6 animals. Data are represented as UMAP and heatmap approximation (A, B), median expression values (C-D), MDS approximations (E) and dot plot expression approximations (F). Statistical comparisons (B-D) were conducted by two-way ANOVA with Sidak’s multiple comparisons test. Significance is defined as p < 0.05.

After identifying shared and tissue-specific TCR sequences, the transcriptional profiles of CM9-specific CD8^+^ T cells with either shared or tissue-specific TCRs were compared across multiple anatomical sites. The largest population of cells with tissue-specific TCR sequences was in the liver, where the T cells with a tissue-specific TCR sequence formed multiple large clusters in the RNA-seq data, while no clear clustering of cells with tissue-specific TCRs were observed in other anatomical sites (Fig 3A). GSEA of cells with shared and tissue-specific TCR sequences identified that none of the Hallmark pathways showed statistically significant differences between cells with shared and tissue-specific TCR sequences (Fig 3B), suggesting CM9 specific CD8^+^ T cells with tissue-specific TCRs do not exhibit significantly different transcriptional profiles when comparing Hallmark gene sets. In an additional GSEA analysis, we evaluated the enrichment scores for the TRM signature, cytotoxic signature, exhausted signature, ISG signature, T_EM_ and T_CM_ signature gene sets as described above. We observed no significant differences in median enrichment values for all the aforementioned gene sets when separated by animal (Fig 3C). However, when separated by anatomical site, cells with a tissue specific TCRs associated with a significantly lower cytotoxic gene enrichment, and ISG gene enrichment (Fig 3D), suggesting cells with tissue specific TCRs are less cytotoxic and less IFN-responsive. Furthermore, as tissue-specific clonotypes did not have differential expression patterns of genes associated with a tissue-resident phenotype, this suggests that additional phenotypic markers may be required to identify these cells in nonhuman primates.

Importantly, a disadvantage of using GSEA is the relatively low number of genes that are being compared between groups of interest, in this case CM9-specific CD8^+^ T cells with shared or tissue specific TCRs. To remedy this, we calculated the average gene expression of all genes that were detected and separated the data by anatomical site and whether the cell expressed a shared or tissue specific TCR. A multi-dimensional scaling (MDS) plot revealed cells with shared and tissue specific TCRs clustered together in most anatomical sites, except for the liver, which saw clear separation of the two populations, suggesting a difference in the transcriptional profile between cells with shared or tissue specific TCRs in the liver. Furthermore, while the lymphoid tissues clustered together, the liver and blood clustered further away, suggesting general transcriptional differences between the blood and other anatomical sites (Fig 3E). We then identified differentially expressed genes in CM9-specific CD8^+^ T cells from PBMCs compared to other anatomical sites to identify the transcriptional signature underlying this clustering. Most of the 20 most differentially expressed genes in cells from the PBMCs were downregulated compared to other anatomical sites, with the exception of apoptosis associated gene, *RASSF3* which had higher expression in cells from the blood (Fig 3F). The genes downregulated in cells from the PBMCs include *ENTPD1*, which encodes CD39, and is known to associate with cellular exhaustion in HIV infection [28]. However, another gene downregulated in CM9-specific CD8^+^ T cells from the blood was *IL21*. HIV-specific IL-21-producing CD8 T cells are enriched in individuals who can control HIV [29], suggesting IL21 expressing cells may contribute to viral control. Downregulation of this gene may indicate a reduced capacity for CM9-specific CD8^+^ T cells from the blood to maintain viral control.

The analysis of T cells with shared and tissue specific TCRs detailed previously only distinguished between those cells with TCRs found in one anatomical site verses those found in more than one anatomical site. To extend this analysis, we compared the RNA-seq profile of cells with TCRs found in 1, 2, 3, 4, or 5 anatomical sites. Using UMAP analysis, we observed no obvious clustering or separation regarding the number of anatomical sites the CM9-specific CD8^+^ T cells are observed (S2A Fig). Similarly, CM9-specific CD8^+^ T cells with TCRs observed in 1–5 anatomical sites were found in all the identified unbiased clusters (S2B Fig), suggesting minimal clustering of cells via the number of anatomical sites in which they are observed. The average gene expression of all genes detected was calculated for CM9-specific CD8^+^ T cells found in 1, 2, 3, 4, or 5 anatomical sites. MDS analysis of these data identified no clear clustering and obvious separation of CM9-specific CD8^+^ T cells found in 1, 2, 3, 4, or 5 anatomical sites (S2C Fig). The MDS analysis suggests a globally distinct transcriptional profile between cells with different degrees of TCR sharing. Lastly, we sought to evaluate if there was a relationship between the expression of TRM marker genes (ITGAE, ITGA1 and CD69) and the number of anatomical sites where a cell is present. There were no significant differences between the average expression of ITGAE, ITGA1 or CD69 between cells observed in 1, 2, 3, 4, or 5 anatomical sites (S2D Fig), suggesting that the expression of TRM marker genes is not predictive of the number of anatomical sites in which the clones reside.

### TCR sequence publicity does not associate with a transcriptional profile

**Fig 4:**
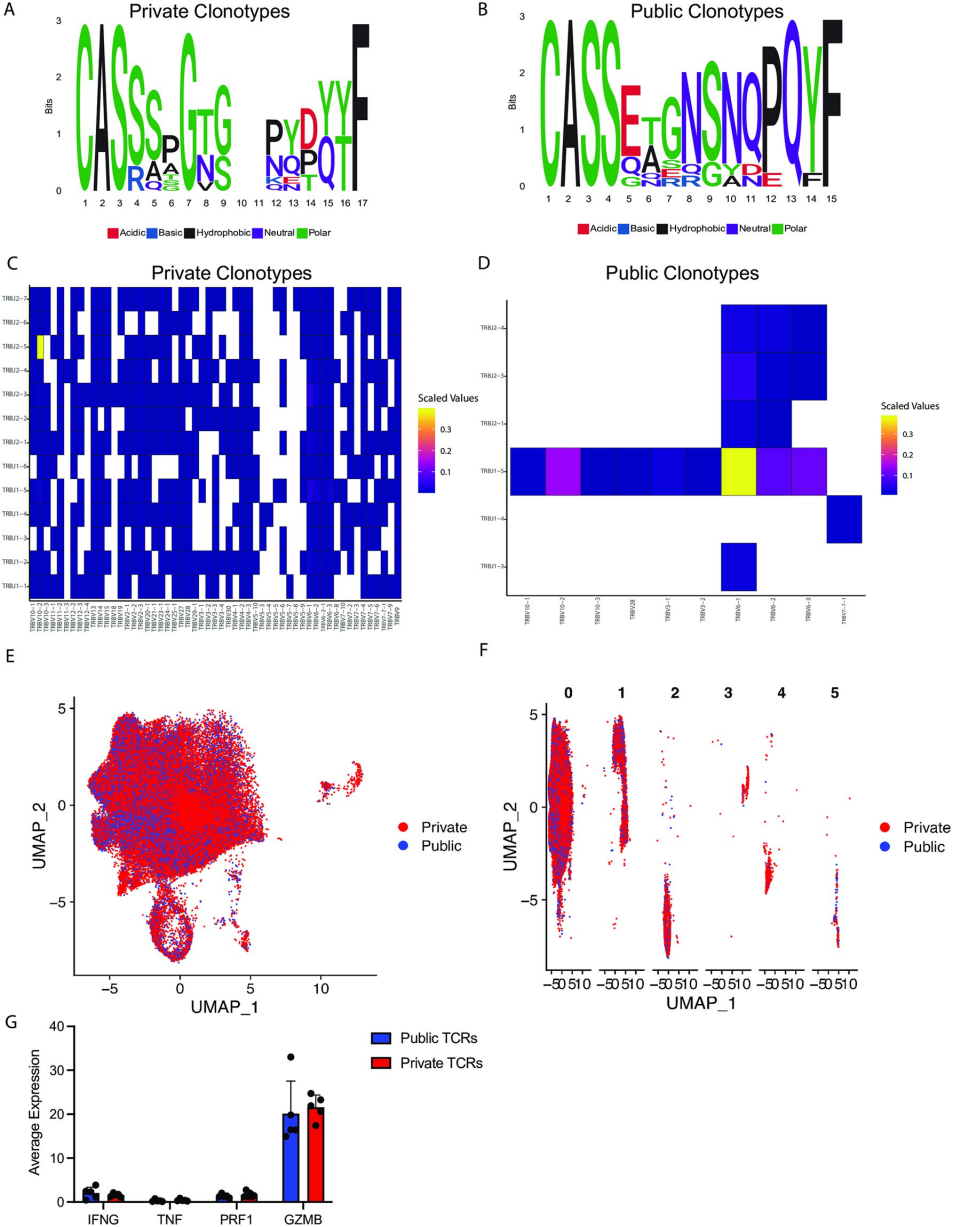
TCR sequence publicity does not associate with a transcriptional profile. (A) Logo plots of the 10 most frequent CDR3 regions in CM9-specific CD8^+^ T cells expressing private clonotypes. (B) Logo plots of the 10 most frequent CDR3 regions in CM9-specific CD8^+^ T cells expressing public clonotypes. (C) Heatmap approximation of the TRB V and J segments in cells with private clonotypes. (D) Heatmap approximation of the TRB V and J segments in cells with public clonotypes. (E) UMAP analysis of RNA-seq data from CM9-specific CD8^+^ T cells from multiple anatomical sites, with color representing cells with public or private clonotypes. (F) UMAP analysis of RNA-seq data from CM9-specific CD8^+^ T cells from multiple anatomical sites, with color representing cells with public or private clonotypes, split by unbiased clustering. (G) Average gene expression of *IFNG*, *TNF*, *PRF1*, and *GZMB*. Data are representative of 6 animals. Data are represented as Logo plots (A, B), heatmap (C, D) and UMAP (E, F) approximations and mean and SD (G). Statistical comparisons (G) were conducted by two-way ANOVAs and Sidak’s multiple comparisons test, with significance defined as p < 0.05.

In addition to evaluating TCR sequences shared between tissues within the same animal, we also sought to determine if there was a relationship between the transcriptional profile of cells with public (expressed in multiple individuals) and private (expressed in only one individual) TCR sequences [30]. Firstly, we examined the public TCR sequences (identified in a total of 12914 cells) compared to private TCR clonotypes (identified in a total of 53650 cells). We identified several differences between the 10 most frequent CDR3 sequences between private and public clonotypes (Fig 4A and 4B). Similarly, V and J segment usage was substantially different between public and private clonotypes, with public clonotypes exhibiting a more restricted repertoire regarding V and J segment usage and an overabundance of TRBV6-1 and TRBJ1-5 compared to private clonotypes (Fig 4C and 4D). These data suggest a select few TCR sequences that favor publicity.

Previous studies have observed a link between a higher number of public TCR clones and viral control in SIV infection [31], raising the question that the publicity of a TCR clone may be associated with a distinct transcriptional profile favorable to viral control. We did not identify any clear clustering with respect to TCR publicity in the transcriptional profile and unbiased clustering of CM9-specific CD8^+^ T cells with clear overlap of cells with public and private TCRs (Fig 4E and 4F). Furthermore, we determined the average expression of gene associated with viral control (*IFNG*, *TNF*, *PRF1*, *GZMB*) and saw no significant differences between CM9-specific CD8^+^ T cells with public or private clonotypes (Fig 4G). These data suggest cells expressing a public TCR do not inherently exhibit a distinct transcriptional profile.

### SIV-specific CD8^+^ T cells from post-treatment controllers exhibit some differences in transcriptional profiles compared to viremic animals

**Fig 5:**
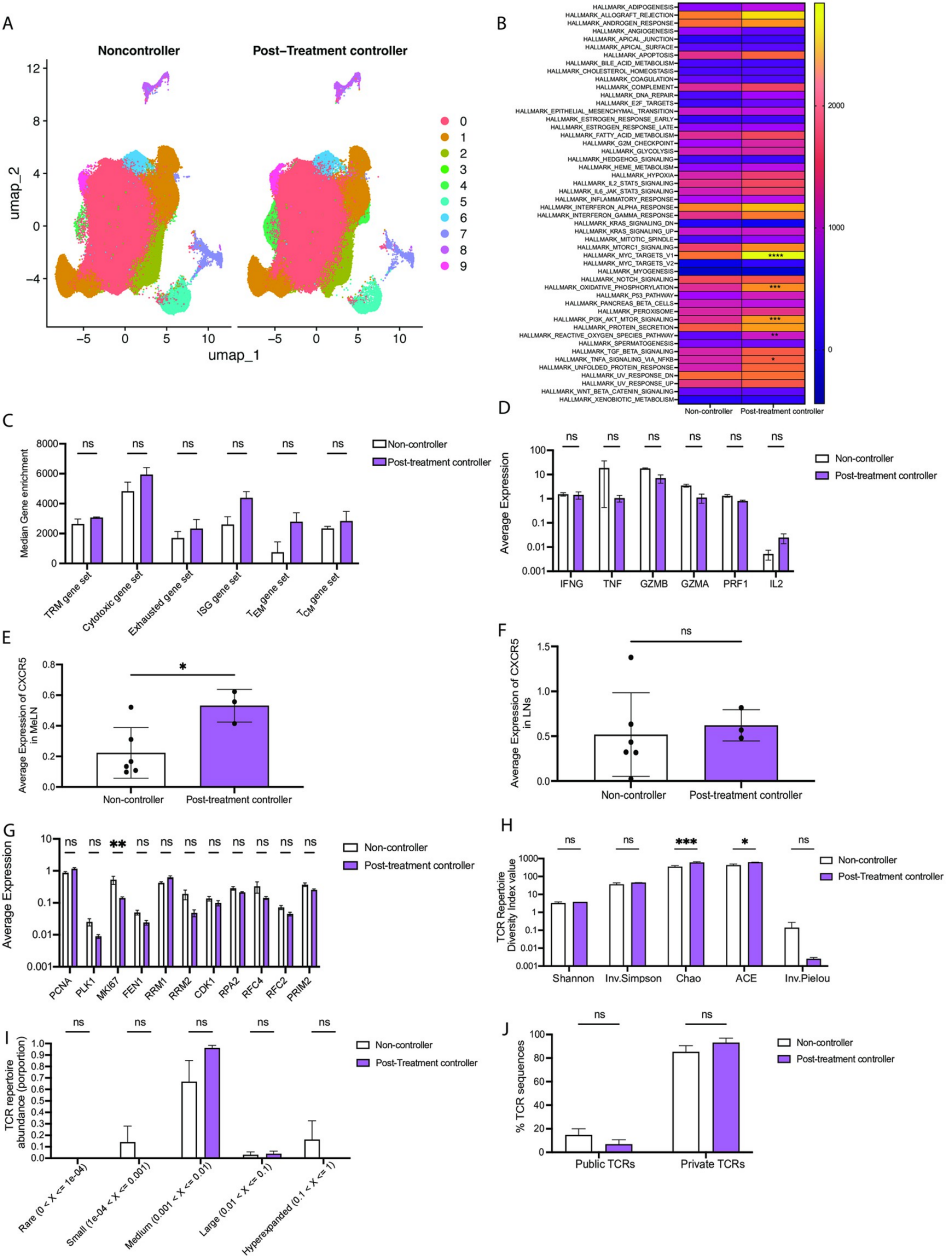
SIV-specific CD8^+^ T cells from post-treatment controllers exhibit some differences in transcriptional profiles compared to cells from viremic animals. Three animals who controlled SIV to low levels (< 200 copies/ml) over 12 months after stopping ARTs were compared to the 6 non-controller animals already assessed. (A) UMAP analysis of RNA-seq data from CM9-specific CD8^+^ T cells from multiple anatomical sites from chronically SIV-infected rhesus macaques, colors designate unbiased clusters, split by controller status. (B) Heatmap analysis of the median expression enrichment value for each of the Hallmark gene sets for non-controllers and post-treatment controllers (down-sample of 10,000 cells for each sample index). (C) Median gene expression levels for the TRM signature gene set (*ITGAE*, *ITGA1*, *RUNX3*, *NR4A1*, *CD69*), the cytotoxic signature gene set (*GZMB*, *GZMA*, *NKG7*, *FCRL6*, *SLAMF7*, *CX3CR1*, *PRF1*), the exhausted signature gene set (*PDCD1*, *LAG3*, *TIGIT*, *CTLA4*, *HAVCR2*), the ISG signature gene set (*IRF7*, *IRF2*, *IRF1*, *MX1*, *BST2*), the T_CM_ gene set (*SELL*, *CCR7*, *TBR1*, *TCF7*, *BCL6*, *ID3*, *STAT3*) and the T_EM_ gene set (*TBX21*, *ID2*, *STAT4*, *KLRG1*) (down-sample of 10,000 cells for each sample index). (D) Averaged expression of genes associated with polyfunctionality. (E) Averaged expression of CXCR5 in CM9-specific CD8^+^ T cells from the MeLN (F) Averaged expression of CXCR5 in CM9-specific CD8^+^ T cells from the LNs. (G) Averaged expression of genes associated with proliferation. (H) TCR repertoire diversity of CM9-specific CD8^+^ T cells as determined by 5 diversity indices. (I) Relative abundance of TCR sequences from CM9-specific CD8^+^ T cells. (J) Percentage of public and private TCR sequences. Data are representative of 9 animals (6 non-controllers, 3 post-treatment controllers). Data are represented as UMAP (A), heatmap (B), as mean and SEM (C-J). Statistical comparisons were conducted by two-way ANOVA with Sidak’s multiple comparisons test (B-D, G-J), or unpaired T-tests (E, F), with significance defined as p < 0.05.

Individuals who can control HIV infection after long-term ART interruption are referred to as post-treatment controllers and these individuals exhibit distinct immunological states compared to non-controllers [32]. We identified three rhesus macaques who retained low viral loads (< 200 copies/ml) for over 100 days after ART cessation and evaluated the transcriptional profile and TCR repertoire of their CM9-specific CD8^+^ T cells (animal information in S1 Table). UMAP analysis of RNAseq data revealed 10 clusters, all of which were present in both non-controllers and post-treatment controllers (Fig 5A), suggesting a lack of global differences in CM9-specific CD8^+^ T cells between the two groups. GSEA using the Hallmark pathways identified 5 pathways that were significantly enriched in the post-treatment controllers (Fig 5B). These pathways were *Myc targets V1*, *oxidative phosphorylation*, *PI3K/AKT/MTOR signaling*, *reactive oxygen species* and *TNFα signaling via NFκB*. GSEA using the TRM signature gene set, the cytotoxic signature gene set, the exhausted signature gene set, the ISG signature gene set, the T_CM_ and T_EM_ gene set showed no significant differences between non-controllers and post-treatment controllers (Fig 5C). Studies involving individuals who can control HIV in the absence of ARTs show their HIV-specific CD8^+^ T cells exhibit enhanced polyfunctionality [1,33] and expression of CXCR5 [34–36], which allows the cells to enter the B-cell follicles to interact with T follicular helper (Tfh) cells within lymphoid tissue, a key reservoir of HIV infection [37]. We sought to determine if a similar phenotype was observed in cells from post-treatment controllers. Firstly, we examined the averaged expression of a number of genes associated with viral control or the immune response to SIV to get a sense of polyfunctionality. These genes included *IFNG*, *TNFA*, *GZMB*, *GZMA*, *PRF1*, *IL2*. None of these genes were differentially expressed between non-controllers and post-treatment controllers (Fig 5D). Post-treatment controllers exhibited significantly higher CXCR5 expression in the MeLN compared to chronically SIV infected animals (Fig 5E), but this was not observed in the other lymph nodes (Fig 5F). Importantly, this was not associated with higher numbers of sorted cells from the MeLN in post-treatment controllers (S4A Fig). We also examined genes associated with cell proliferation, to determine if cells from post-treatment controllers were more proliferative, but again saw no significant differences between non-controllers and post-treatment controllers, with the exception of a significantly lower expression of *MKI67* in the post-treatment controllers (Fig 5G). Together, these data suggest that while several specific genes and pathways may differ between non-controllers and post-treatment controllers, the overall transcriptional profiles of CM9-specific CD8^+^ T cells from these groups are largely similar.

We also sought to examine if the TCR repertoires of CM9-specific CD8^+^ T cells from non-controllers and post-treatment controllers were distinct. Firstly, we determined the diversity of the TCR repertoire using 5 different diversity measures. CM9-specific CD8^+^ T cells from post-treatment controllers exhibited significantly higher Chao and ACE scores compared to non-controllers where all other diversity indices were not significant (Fig 5H), suggesting a slightly more diverse TCR repertoire in CM9 specific CD8^+^ T cells from post-treatment controllers. The TCR repertoire constitution was analyzed, and no differences were observed between CM9 specific CD8^+^ T cells from non-controllers and post-treatment controllers, with the repertoire being largely oligoclonal for both groups (Fig 5I). Lastly, as previous studies had observed an association between viral control and a higher number of public clonotypes [31,38], we sought to identify the frequency of public and private TCR sequences in the viremic animals and post-treatment controllers. We observed no significant differences between viremic animals and post-treatment controllers regarding public or private clonotype usage (Fig 5J). Overall, the TCR repertoires of CM9 specific CD8^+^ T cells from non-controllers and post-treatment controllers were similar.

### SIV-specific CD8^+^ T cells show some differences in transcriptional profiles compared to CMV-specific CD8^+^ T cells

**Fig 6:**
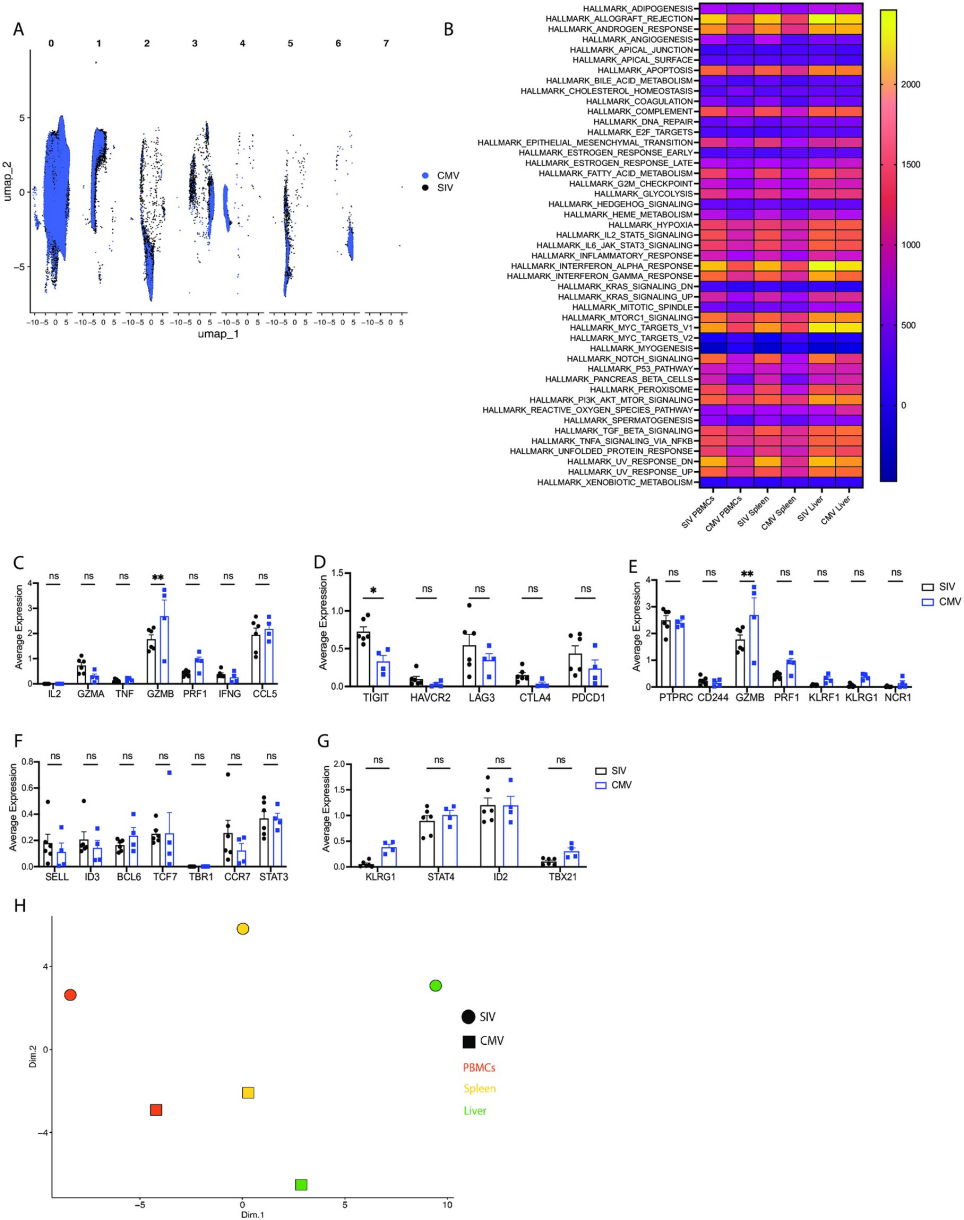
CMV and SIV-specific CD8^+^ T cells exhibit some differences in transcriptional phenotype. (A) UMAP analysis of RNA-seq data from SIV CM9 and CMV VY9-specific CD8^+^ T cells from multiple anatomical sites from chronically SIV and CMV infected rhesus macaques, colors designate cells antigen specificity, separated by unbiased clusters. (B) Heatmap showing median expression enrichment value for each of the Hallmark gene sets for each anatomical site. (C) Average gene expression for genes associated with cytotoxicity or cytokine expression. (D) Average gene expression for genes associated with exhaustion. (E) Average gene expression for genes associated with TERMA cells. (F) Average gene expression for genes associated with T_CM_ cells. (G) Average gene expression for genes associated with T_EM_ cells. (H) Multidimensional scaling (MDS) analysis of the average gene expression of all genes in cells specific for either SIV or CMV for each anatomical site. Data are representative of 6 animals (SIV) and 4 animals (CMV). Data are represented as UMAP (A), heatmap (B) or MDS approximations (H) or as mean and SD (C-G). Statistical comparisons (B-G) were conducted by two-way ANOVAs with Sidak’s multiple comparisons test. Significance is defined as p < 0.05.

We sought to compare the RNA-seq profile of CM9-specific CD8^+^ T cells to cells specific for another epitope associated with chronic viral infection. As the rhesus macaques in our study had been naturally infected with rhesus CMV, we utilized an MHC Class I Pentamer containing the CMV epitope VY9 (VTTLGMALY) to identify CMV-specific CD8^+^ T cells [39]. UMAP analysis revealed minimal clustering by antigen (Fig 6A) and no significant differences were observed using GSEA of the Hallmark pathway gene sets between SIV CM9-specific CD8^+^ T cells and CMV VY9-specific CD8^+^ T cells (Fig 6B), suggesting generally similar transcriptional profiles. Comparisons of the TCR repertoire diversity and abundance revealed CMV VY9-specific CD8^+^ T cells exhibit a repertoire with increased sequence abundance, as defined by the Chao index, but reduced richness, as defined by the ACE diversity score (S3A Fig). CMV VY9-specific CD8^+^ T cells had a significantly higher proportion of large clonotypes compared to SIV-specific CD8^+^ T cells (S3B Fig).

Previous studies have identified enhanced cytokine and cytotoxic responses, and reduced exhaustion makers in CMV-specific CD8^+^ T cells compared to SIV-specific CD8^+^ T cells [39]. This phenotype was associated with expanded terminally differentiated effector memory (TEMRA) cells [39], known to upregulate genes such as *PTPRC*, *CD244*, *GZMB*, *PRF1*, *KLRF1*, *KLRG1*, *NCR1* [40]. We therefore assessed the expression of cytokine genes and genes associated with cytotoxicity, exhaustion, and TEMRA cells. Significant differences in gene expression were observed only in *GZMB* expression regarding genes associated with cytotoxicity/cytokine response (Fig 6C), and only in *TIGIT* regarding genes associated with exhaustion (Fig 6D). *GZMB* expression is also associated with a TEMRA phenotype, but no other genes associated with TEMRA cells were significantly elevated in CMV-specific cells (Fig 6E). Genes associated with T_CM_ cells and T_EM_ cells were also not significantly different between CMV and SIV-specific CD8^+^ T cells (Fig 6F and 6G). Together, these analyses suggest some minor increases in cytotoxicity and decreases in exhaustion in CMV-specific CD8^+^ T cells compared to SIV-specific CD8^+^ T cells. Finally, MDS analysis of the average gene expression of all genes showed vertical separation of SIV and CMV-specific CD8^+^ T cells (Fig 6F), suggesting a general distinction in RNA-seq profiles between cells specific for different antigens.

## Discussion

In this study, we assessed the relationship between the transcriptional profile and the anatomical location and TCR sequences of SIV CM9-specific and CMV VY9-specific CD8^+^ T cells. We identified minimal differences in transcriptional profiles between CM9-specific CD8^+^ T cells from different anatomical sites. While there was downregulation of select pathways in cells found in only one anatomical site, there were generally similar transcriptional profiles between CM9-specific CD8^+^ T cells found in one anatomical site verses more than one site. Similarly, no obvious differences between the transcriptional profile of cells with public or private TCRs were identified. Comparisons between CM9 specific CD8^+^ T cells from post-treatment controllers and chronically SIV-infected animals revealed an upregulation of pathways affiliated with activation and higher CXCR5 expression in the MeLN associating with post-treatment control. Finally, MDS comparisons between CM9-specific CD8^+^ T cells and VY9-specific CD8^+^ T cells showed distinct transcriptional profiles, suggesting a general difference in phenotype in cells with different antigen specificities.

Lack of significant transcriptional alterations of CM9-specific CD8^+^ T cells across multiple anatomic sites suggests that upon infection, antigen specific CD8^+^ T cell expansion seeds multiple anatomical sites, some of which are maintained throughout chronic infection. Indeed, we have previously observed clonal persistence of CM9-specific CD8^+^ T cells in distinct anatomical sites from acute infection through chronic infection [12]. While the local microenvironment affecting these cell populations may be sufficient to induce some general alterations to transcription, as evidenced by MDS analysis, it is not sufficient to induce significantly distinct phenotypes. It must be noted however, that the cell input was not equal across the different anatomical sites, specifically in the liver, where the cell number was lower than other sites. This uneven cell input may have limited our ability to detect differences between anatomical sites. Moreover, as sequence depth and cell capture procedures for scRNAseq continue to improve, it’s possible that future analysis will unravel modest transcriptional differences we were unable to identify. Indeed, we also observed no relationship between the TCR sequence and the transcriptional profile. While previous reports have linked TCR sequence to cell phenotypes [41,42], these studies included total CD8^+^ T cells and the observed relationships were associated with antigen specificity. In our study, where all cells are specific for the same epitope, there was no obvious relationship between TCR sequence and phenotype, which may explain the discrepancy. We believe it is unlikely that differential mutation of the CM9 epitope dramatically contributed to our findings. We have previously sequenced the epitope in plasma from a large cohort of animals and found very little mutation away from the wild-type sequence [43]. Moreover, we have also previously found minimal differences between epitope recognition by T cells with public or private TCR clonotypes [31]. Exploration of transcriptional profiles of individual subsets of antigen-presenting cells which present the CM9 epitope may also help understand CD8 T cell responses.

Previous studies identified CM9 specific CD8^+^ T cells with tissue specific TCRs sequences during chronic SIV infection [12,13]. We evaluated the transcriptional profile of cells with shared or tissue specific TCR sequences and observed significantly lower expression of cytotoxic and ISG genes in cells with tissue specific TCRs compared to cells with shared TCRs within the same anatomical site. However, we observed no association between the transcriptional phenotype and the number of anatomical sites a TCR was sampled. These data suggest that, firstly, within each anatomical site, there are distinct populations of cells that have recently been circulating and those that are residing in that one anatomical site, however, this residing population was not found to associate with genes associated with tissue residency. Indeed, it has previously been speculated that not all TRMs express the canonical markers CD103, CD49a and CD69 [44], especially CD103 in secondary lymphoid tissue [45], and it is possible that analysis of less canonical markers may characterize cells with tissue specific TCRs. Furthermore, given the overrepresentation of secondary lymphoid tissue in this study, we questioned whether the tissue specificity of the cells associated with T_EM_ or T_CM_ phenotypes, but observed no association between cells with shared and tissue specific TCRs and their memory subtype. The distinction between T cells with tissue specific and shared TCRs was clearly observed in the liver using MDS analysis, possibly suggesting reduced trafficking to the liver compared to secondary lymphoid tissue. Alternatively, it is possible the tissue specific CM9-specific CD8^+^ T cells exhibited extensive local proliferation in intrahepatic myeloid-cell aggregates for T cell population expansion (iMATE) [46,47], creating a distinct microenvironment and subsequent transcriptional profile.

Secondly, our data illustrate that CM9 specific CD8^+^ T cells found in one anatomical site are less transcriptionally active regarding pathways associated with cytotoxicity and ISG signaling than those found in multiple sites. This suggests that recently circulating cells, or clones that have persisted in multiple tissues since acute infection, are more capable of controlling infection through higher cytotoxicity, although further analysis of these populations is required to confirm this hypothesis.

In addition to analyzing CM9 specific CD8^+^ T cells from chronically SIV infected animals, we also sought the characterize the transcriptional profile in cells from post-treatment controllers. Little is known about the transcriptional profile of HIV specific CD8^+^ T cells from individuals who control HIV following treatment interruption. However, multiple studies of HIV specific CD8^+^ T cells from elite controllers have associated several characteristics with viral control, including enhanced proliferation [48,49], enhanced cytotoxicity and cytokine production [1,33], and increased expression of CXCR5 to allow for killing of Tfh cells [34–36]. We therefore assessed these phenotypes in the post-treatment controllers. While the overall RNA-seq profile of CM9 specific CD8^+^ T cells from both chronically infected and post-treatment controllers showed similar clustering, several Hallmark pathways associated with cellular activation (oxidative phosphorylation [50] and reactive oxygen species [51]) or cytokine signaling (TNFα signaling via NFκB) were elevated in the T cells from the controllers. This suggests an enhanced functional phenotype in antigen specific CD8^+^ T cells in post-treatment controllers. However, elevated expression of cytokines or genes associated with proliferation were not present in cells from post-treatment controllers. Finally, we observed a significantly higher expression of CXCR5 in the CM9-specific CD8^+^ T cells from the MeLN in the post-treatment controllers. As this phenotype was not observed in the other lymph nodes assessed, it suggests a disproportionate role for the MeLN as an important reservoir of SIV, supported by previous studies identifying higher SIV DNA in the MeLN compared to blood and other lymph nodes [52]. Furthermore, these data suggest a lack of follicle-homing CD8^+^ T cells in the MeLN represents a significant barrier to viral control.

Our final series of analyses compared the transcriptional profile of SIV CM9-specific CD8^+^ T cells to CMV VY9-specific CD8^+^ T cells to identify if distinct transcriptomic phenotypes are associated with epitopes from different viruses that induce a chronic infection in the host. We observed clear separation by epitope in the average gene expression MDS analysis, suggesting distinct phenotypes in T cells with distinct epitope specificities, similar to previous analyses with CD8^+^ T cells specific for CMV, Epstein Barr virus, and influenza virus [53]. Unlike SIV-specific CD8^+^ T cells which exhibit signs of functional exhaustion in chronic infection [26,54], CMV-specific CD8^+^ T cells are known to be somewhat resistant to an exhaustion phenotype [55–58]. Indeed, direct comparisons between CMV and SIV-specific CD8^+^ T cells in a previous study involving rhesus macaques showed reduced exhaustion-associated protein expression in CMV- specific cells [39]. However, we observed only 2 differentially expressed genes between CMV and SIV-specific CD8^+^ T cells; elevated *GZMB* and reduced *TIGIT* expression in CMV-specific T cells. It is unclear if the altered expression of only 2 genes is sufficient to represent higher cytotoxicity and lower exhaustion in CMV-specific CD8^+^ T cells compared to SIV-specific CD8^+^ T cells. This lack of statistical significance across multiple genes may be indicative of an under-powered study, as the CMV specific CD8^+^ T cells represent only 4 animals. Alternatively, the lack of decreased cellular exhaustion may associate with a lack of multiple upregulated genes associated with TEMRA cells in CMV specific CD8^+^ T cells compared to SIV specific CD8^+^ T cells in this study. These data suggest a lack of TEMRA expansion in our animals, a cell phenotype previously observed concurrently with a lack of exhaustion makers in CMV specific CD8^+^ T cells [39,59].

In this study, we utilized single-cell multi-omics to determine the TCR repertoire and transcriptional profile of antigen specific CD8^+^ T cells in multiple anatomical sites in chronically SIV and CMV infected rhesus macaques. We identified SIV-specific CD8^+^ T cells exhibit similar phenotypes in different anatomical sites but show some distinct pathway enrichment between cells with shared or tissue specific TCRs within each anatomical site. Further analysis revealed enrichment of pathways associated with cellular activation in CM9-specific CD8^+^ T cells from post-treatment controllers and identified a broad difference in transcriptional profiles between cells specific for SIV and CMV. Together, these data constitute an in-depth analysis of antigen specific CD8^+^ T cells across multiple anatomical sites, explore the relationship between TCR sequence and phenotype and provide mechanistic insight into tissue-specific T cell immunity and antiviral activity. These data can be used as a framework upon which to develop therapeutic interventions aimed at inducing tissue-specific T cell immunity.

## Materials and methods

### Ethics statement

The National Institute of Allergy and Infectious Diseases (NIAID) animal care and use committee, as part of the National Institute of Health (NIH) intramural research program, approved all experimental procedures pertaining to the animals (protocol LVD 26E). The animals in this study were housed and cared for at the NIH animal center, under the supervision of the Association for the Assessment and Accreditation of Laboratory Animal Care (AAALAC)-accredited division of veterinary resources and as recommended by the office of animal care and use nonhuman primate management plan. Care at this facility met the standards set forth by the animal welfare act, animal welfare regulations, United States fish and wildlife services regulations, as well as the guide for the care and use of laboratory animals (8th Edition). The physical conditions of the animals were monitored daily. Animals in this study were exempt from contact social housing due to scientific justification, per respective the NIAID/NIH institutional animal care and use committee (IACUC) protocol, and were housed in non-contact, social housing where primary enclosures consisted of stainless-steel primate caging. The animals were provided continuous access to water and offered commercial monkey biscuits twice daily as well as fresh produce, eggs and bread products and a foraging mix consisting of raisins, nuts, and rice. Enrichment to stimulate foraging and play activity was provided in the form of food puzzles, toys, cage furniture, and mirrors.

### Animals

Nine Mamu-A*01^+^ rhesus macaques (*Macaca mulatta*) were infected intravenously with 3,000 TCID_50_ of SIVmac239 and necropsied during chronic infection. Four Mamu-A*02^+^ rhesus macaques that were naturally infected with CMV were also necropsied after infection. Three animals in this study met our criteria for post-treatment SIV controllers. These animals controlled SIV viral load to fewer than 200 copies/ml plasma for over 12 months after receiving daily coformulated subcutaneous injections of the nucleo(s/t)ide reverse transcriptase inhibitors Emtricitabine (FTC) and Tenofovir Disoproxil Fumarate (TDF), a prodrug of Tenofovir (TFV), and the integrase strand-transfer inhibitor Dolutegravir (DTG). All animals were male and other details are included in S1 Table. PBMCs, LNs, MeLN, spleen, liver, and gut (jejunum and colon) were taken from each animal. Single-cell suspensions from PBMCs and processed tissue biopsies were generated and used for flow cytometric sorting.

### Flow cytometric sorting

Single-cell suspensions were washed twice with PBS (HyClone). SIV or CMV-specific CD8^+^ T cells were identified by fluorochrome-conjugated CM9 (CTPYDINQM) or VY9 (VTTLGMALY) MHC Class I Pentamers (Proimmune). Antibodies against cell surface markers utilized to identify lymphocytes are detailed in S2 Table. Dead cells were excluded using Live/dead Aqua dead cell stain kit (ThermoFisher). CM9 or VY9-specific CD8^+^ T cells were sorted using an S6 Symphony Cell Sorter (BD). Total number of cells sorted/cell inputs for sequencing are quantified in S4A Fig. Primary gating strategy for cell sorting is displayed in S4B Fig.

### Multi-omics single cell library generation

Sorted CM9-specific CD8^+^ T cells were then placed into a 10x Genomics Chromium head unit (10x Genomics) according to manufacturers’ instructions. The resulting samples were then processed using the Chromium Next GEM Single Cell V(D)J Reagent Kits v1.1 or v2. Generation of the gene expression library were performed according to the manufacturers’ protocol. The VDJ libraries were also generated according to the manufacturers’ protocol, except for the substitution of rhesus macaque specific TCR C region primers in place of the human primers. For the v1.1 reagents, the first TCR enrichment PCR utilized the forward primer 5’- AATGATACGGCGACCACCGAGATCTACACTCTTTCCCTACACGACGCTC-3’ (final concentration of 1μM) and reverse primers 5’- GTCTGCTGGAATAACGCTGTCC-3’ (final concentration of 1μM) and 5’-GCGCTGATCTTTTGGGTGATGG-3’ (final concentration of 0.5μM) targeting the α and β constant regions respectively. The second TCR enrichment PCR utilized the forward primer 5’-AATGATACGGCGACCACCGAGATCT-3’ (final concentration of 1μM) and the reverse primers 5’- ATGCACGTCAGAATCCTTGC-3’ (final concentration of 1μM) and 5’- CAGAAGGTGGCCGAGACC-3’ (final concentration of 0.5μM) targeting the α and β constant regions respectively. Both enrichment PCRs utilized the cycling conditions of 98°C for 45 seconds, 12 cycles of 98°C for 20 seconds, 60°C for 30 seconds and 72°C for 1 minute, followed by 72°C for 1 minute. A lid temperature 105°C was used for both enrichment PCRs.

For the v.2 10x reagents, the primers utilized for the first enrichment PCR were 5’-GATCTACACTCTTTCCCTACACGACGC-3’ (final concentration of 0.125μM) with reverse primers 5’- CGGCCACTTTCAGGAGGAG-3’ and 5’- CCCACTCACCTGCTCTACC-3’ (final concentration of 1μM for both). The second enrichment PCR utilized the forward primer 5’-GATCTACACTCTTTCCCTACACGACGC-3’ (final concentration of 0.125μM) and reverse primers 5’-TGTCTGTGATATGCACGTCAGA-3’ and 5’-TCAAACACAGCGACCTTGGG-3’ (final concentration of 1μM for both). Both enrichment PCRs utilized the cycling conditions of 98°C for 45 seconds, 20 cycles of 98°C for 20 seconds, 62°C for 30 seconds and 72°C for 1 minute, followed by 72°C for 1 minute. A lid temperature 105°C was used for both enrichment PCRs.

### Sequencing

The single cell libraries were pooled in relation to the number of loaded cells prior to sequencing. Sequencing of the libraries occurred on NovaSeq or NextSeq sequencing machines (Illumina) for both RNA-seq and TCR-seq libraries. Target numbers of reads per cell were 50,000 and 10,000 for gene expression libraries and TCR enriched libraries respectively. For the libraries prepared with the 10x Genomics v1.1 kits, the sequencing parameters were Read 1: 26 cycles, i7 Index: 8 cycles, i5 Index: 0 cycles, and Read 2: 91 cycles. For the libraries prepared with the 10x Genomics v2 kits, the sequencing parameters were Read 1: 26 cycles, i7 Index: 10 cycles, i5 Index: 10 cycles, and Read 2: 90 cycles.

### Sequencing analysis

Initial sequencing analysis utilized *CellRanger* (10x Genomics) pipelines to identify TCR sequences and gene reads. Sample QC and integration of data from different anatomical sites/animals was conducted in the R package *Seurat* (5.0.0) [60], utilizing the QC and integration workflows. The integration method used integration anchors and the rpca reduction. Analysis and identification of clonotypes was performed with the R package *scRepertoire* (1.11.0) in conjunction with *Seurat*. All analyses and visualizations were conducted with *Seurat* pipelines, with the exception of the gene set enrichment analysis (GSEA) which was completed using the R package *Escape* in conjunction with *Seurat* objects.

### Statistical analysis

Statistical analyses were conducted in *Seurat* and *Escape* R packages. Confirmational statistical tests and comparisons between animals and tissues were performed in Prism 9.3.1. Details of statistical analyses for data in each figure are included in the figure legends.

## Supporting information

S1 Table Animal information. (DOCX)

S2 Table Antibodies used for cell sorting. (DOCX)

S1 Fig Characterization of unbiased clusters in CM9-specific CD8^+^ T cells from SIV-infected rhesus macaques. (A) Heatmap analysis of unbiased clustering (down-sample of 10,000 cells per sample index). (B) The percentage of total cells from each anatomical site identified in each cluster. (C) The percentage of total cells from each anatomical site identified in clusters 0 and 1. (D) Heatmap analysis of unbiased clustering from clusters 0 and 1. Data are representative of 6 animals. Data are represented as heatmap approximations (A, D) and individual values (B, C).
(TIF)

S2 Fig No differences observed in the transcriptional profile of CM9-specific CD8^+^ T cells with TCRs shared between 1 and 5 anatomical sites. (A) UMAP analysis of RNA-seq data, with colors signifying the number of anatomical sites in which each TCR is observed. (B) UMAP analysis of RNA-seq data, with colors signifying the number of anatomical sites in which each TCR is observed, separated by unbiased clustering. (C) MDS plot of the average expression of all genes, with points denoting the number of anatomical sites each TCR is observed. (D) Average expression of TRM-associated genes *ITGAE*, *ITGA1* and *CD69*. Data are representative of 6 animals. Data are represented as UMAP (A-B) and MDS approximations (C) in addition to mean and individual values (D). Statistical comparisons for (D) were achieved via 2-way ANOVAs with Tukey’s multiple comparisons test (D), with significance defined as p < 0.05.
(TIF)

S3 Fig TCR repertoire analysis of SIV CM9 and CMV VY9-specific CD8^+^ T cells in multiple anatomical sites. (A) Diversity index values for SIV CM9 and CMV VY9-specific CD8^+^ T cells in all anatomical sites. (B) Relative abundance of rare TCR sequences (0 < X < = 1e−04), small (low occurrence) TCR sequences (1e−04 < X < = 0.001), medium TCR sequences (0.001 < X < = 0.01), large TCR sequences (0.01 < X < = 0.1) and hyperexpanded TCR sequences (0.1 < X < = 1). Data are representative of 6 animals (CM9) and 4 animals (VY9). Data are represented as mean with SD. Statistical comparisons were achieved via 2-way ANOVAs with Sidak’s multiple comparisons test, with significance defined as p < 0.05.
(TIF)

S4 Fig Cell input numbers and gating strategy for cell sorting. (A) Total number of sorted SIV CM9 or CMV VY9-specific CD8^+^ T cells from all animals in each study. (B) Gating strategy for sorting SIV CM9 or CMV VY9-specific CD8^+^ T cells in all anatomical sites. Data are represented as individual values and means (A) and flow plots (B). Statistical comparisons (A) were achieved via multiple effects analysis with Tukey’s multiple comparisons test, with significance defined as p < 0.05.
(TIF)
