# Supplementary material for: Multi-omics analysis of SIV-specific CD8+ T cells in multiple anatomical sites
Source: PLoS Pathog. 2024 Sep 9;20(9):e1012545. doi: 10.1371/journal.ppat.1012545 (PMC11412524; doi:10.1371/journal.ppat.1012545)
Supplement: S1 Table — (DOCX) [file ppat.1012545.s001.docx]

**S1_Table: Animal information**

| Animal ID | Mamu  Genotype | Epitope | Viral load (copies/ml) | Controller Status | Days Post Infection (DPI) | ARV initiation DPI | # days on ARVs | # days post ARV treatment | Anatomical Sites Obtained |
| --- | --- | --- | --- | --- | --- | --- | --- | --- | --- |
| DFH4 | A*01*^+^* | CM9 (SIV) | 1,200,000 | Non-controller | 581 | NA | NA | NA | PBMCs, spleen, LNs, MeLN, liver |
| DBJK | A*01*^+^* | CM9 (SIV) | 1,100,000 | Non-controller | 287 | NA | NA | NA | LNs, MeLN, liver |
| DGXD | A*01*^+^* | CM9 (SIV) | 130,000 | Non-controller | 639 | NA | NA | NA | PBMCs, spleen, LNs, MeLN |
| DGRV | A*01*^+^* | CM9 (SIV) | 73,000 | Non-controller | 699 | NA | NA | NA | PBMCs, spleen, LNs, MeLN, liver |
| DGXJ | A*01*^+^* | CM9 (SIV) | 650,000 | Non-controller | 905 | NA | NA | NA | PBMCs, spleen, LNs, MeLN, liver |
| DG3H | A*01*^+^* | CM9 (SIV) | 2,100,000 | Non-controller | 877 | NA | NA | NA | PBMCs, spleen, LNs, MeLN, liver |
| DGPZ | A*01*^+^* | CM9 (SIV) | 40 | Post-treatment controller | 905 | 117 | 208 | 508 | PBMCs, spleen, LNs, MeLN |
| DGP2 | A*01*^+^* | CM9 (SIV) | 110 | Post-treatment controller | 1031 | 404 | 104 | 487 | PBMCs, spleen, LNs, MeLN, gut |
| DGRA | A*01*^+^* | CM9 (SIV) | 15 | Post-treatment controller | 883 | 467 | 140 | 276 | PBMCs, spleen, LNs, MeLN, gut |
| DF86 | A*02*^+^* | VY9 (CMV) | NA | NA | Natural Infection | NA | NA | NA | PBMCs, spleen, liver |
| DFT1 | A*02*^+^* | VY9 (CMV) | NA | NA | Natural Infection | NA | NA | NA | PBMCs, spleen |
| H46K | A*02*^+^* | VY9 (CMV) | NA | NA | Natural Infection | NA | NA | NA | PBMCs, spleen |
| CB5X | A*02*^+^* | VY9 (CMV) | NA | NA | Natural Infection | NA | NA | NA | PBMCs, spleen |
