# Supplementary material for: Multi-omics analysis of SIV-specific CD8+ T cells in multiple anatomical sites
Source: PLoS Pathog. 2024 Sep 9;20(9):e1012545. doi: 10.1371/journal.ppat.1012545 (PMC11412524; doi:10.1371/journal.ppat.1012545)
Supplement: S2 Table — (DOCX) [file ppat.1012545.s002.docx]

**S2_Table: Antibodies used for cell sorting.**

| Antigen | Fluorochrome | Clone | Supplier | Catalog ID |
| --- | --- | --- | --- | --- |
| CD8 | Pacific Blue | RPA-T8 | BD Biosciences | 558207 |
| NKG2a | PE | Z199 | Beckman Coulter | 1M3291U |
| CD3 | PerCP-Cy5.5 | SP34-2 | BD Biosciences | 552852 |
| CD4 | BV650 | OKT4 | BioLegend | 317436 |
| CD45 | BV786 | D058-1283 | BD Biosciences | 563861 |
| CD20 | APC-H7 | 2H7 | BD Biosciences | 560734 |
| CD11b | BV650 | ICRF44 | BioLegend | 301325 |
| Live/Dead | Aqua Blue | N/A | Thermo Fisher Scientific | L34957 |
