# Supplementary figures and images for: Multi-omics analysis of SIV-specific CD8+ T cells in multiple anatomical sites
Source: PLoS Pathog. 2024 Sep 9;20(9):e1012545. doi: 10.1371/journal.ppat.1012545 (PMC11412524; doi:10.1371/journal.ppat.1012545)

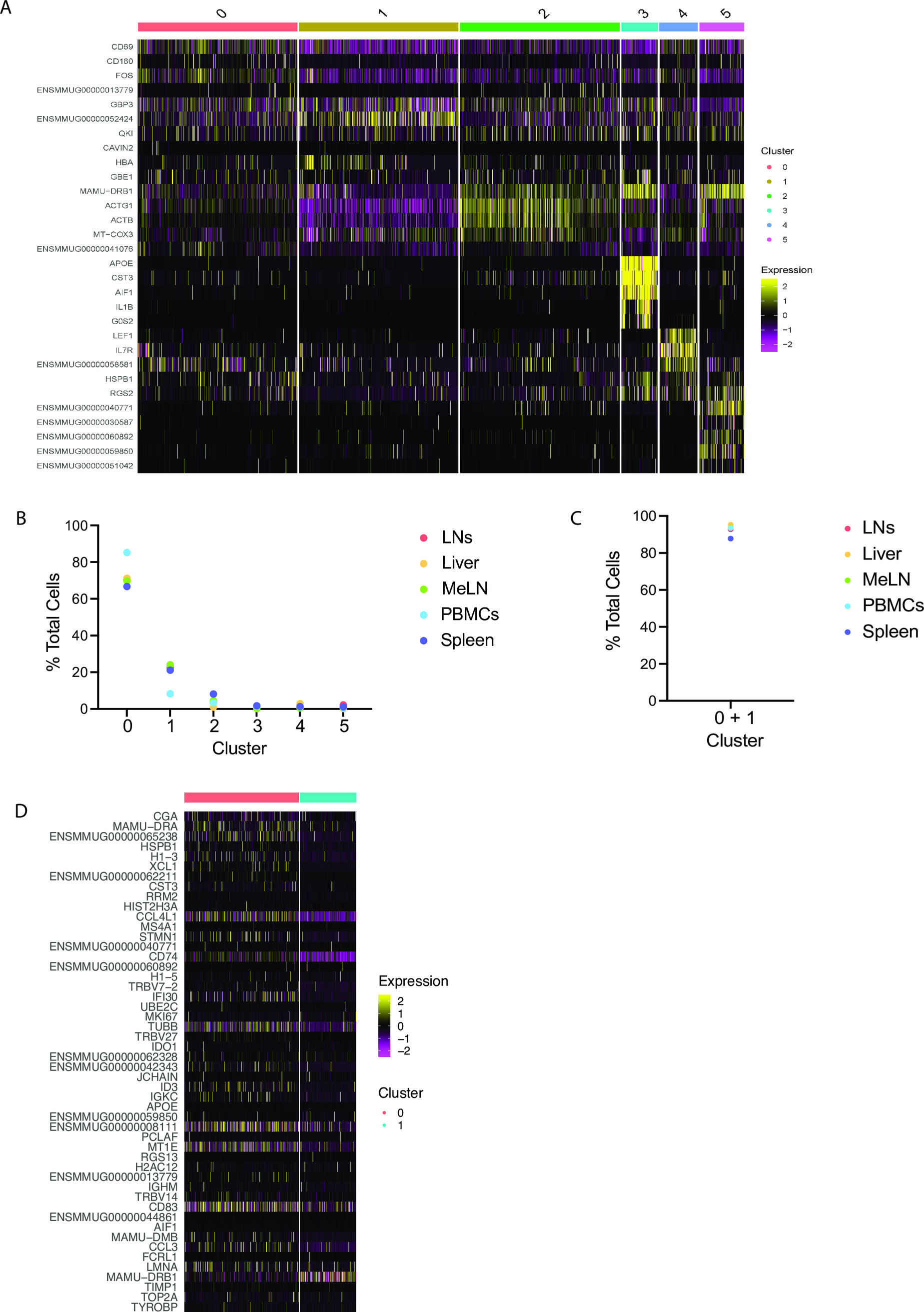

Supplement: S1 Fig — (A) Heatmap analysis of unbiased clustering (down-sample of 10,000 cells per sample index). (B) The percentage of total cells from each anatomical site identified in each cluster. (C) The percentage of total cells from each anatomical site identified in clusters 0 and 1. (D) Heatmap analysis of unbiased clustering from clusters 0 and 1. Data are representative of 6 animals. Data are represented as heatmap approximations (A, D) and individual values (B, C). (TIF) [file ppat.1012545.s003.tif]

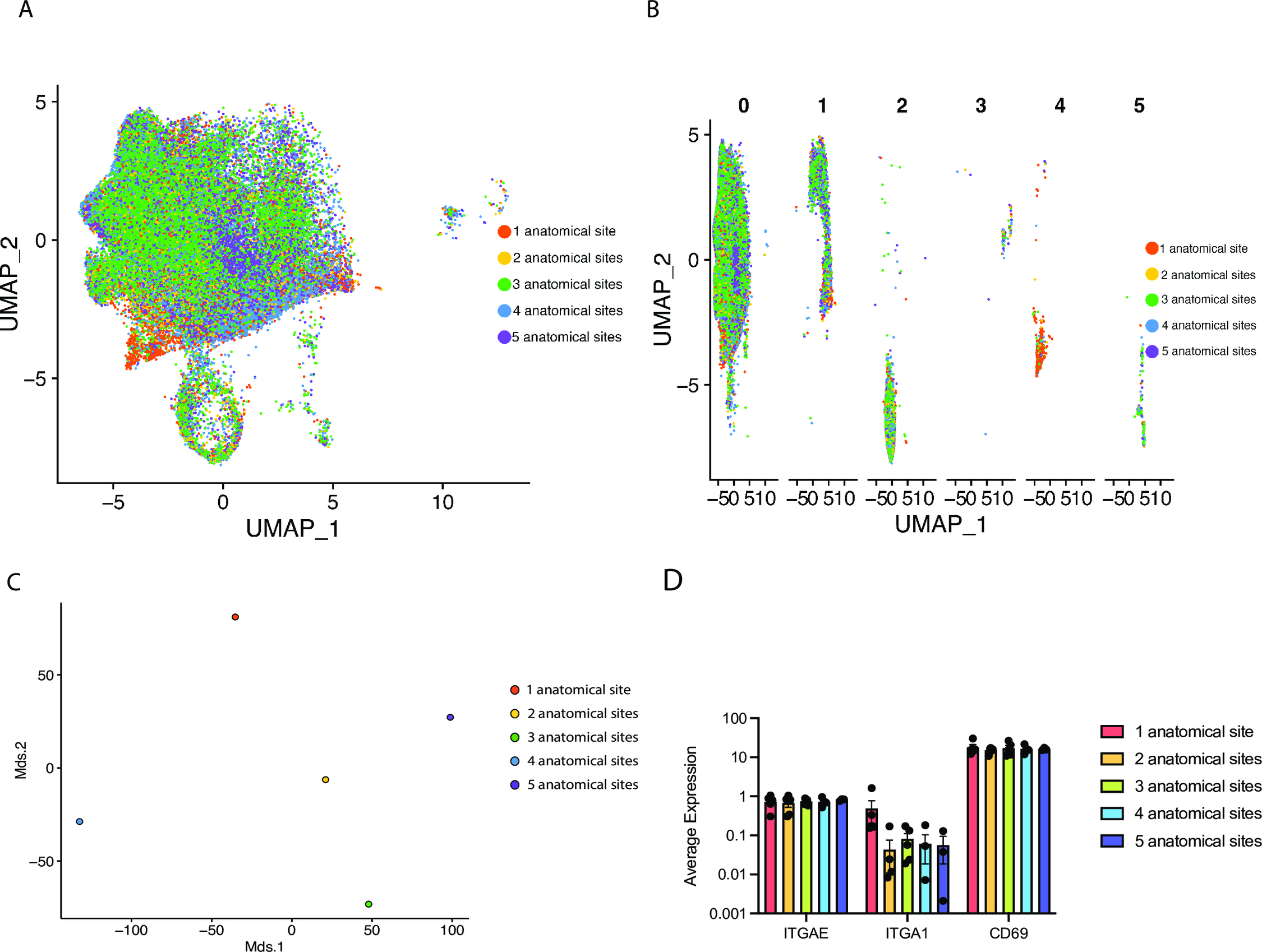

Supplement: S2 Fig — (A) UMAP analysis of RNA-seq data, with colors signifying the number of anatomical sites in which each TCR is observed. (B) UMAP analysis of RNA-seq data, with colors signifying the number of anatomical sites in which each TCR is observed, separated by unbiased clustering. (C) MDS plot of the average expression of all genes, with points denoting the number of anatomical sites each TCR is observed. (D) Average expression of TRM-associated genes ITGAE, ITGA1 and CD69. Data are representative of 6 animals. Data are represented as UMAP (A-B) and MDS approximations (C) in addition to mean and individual values (D). Statistical comparisons for (D) were achieved via 2-way ANOVAs with Tukey’s multiple comparisons test (D), with significance defined as p < 0.05. (TIF) [file ppat.1012545.s004.tif]

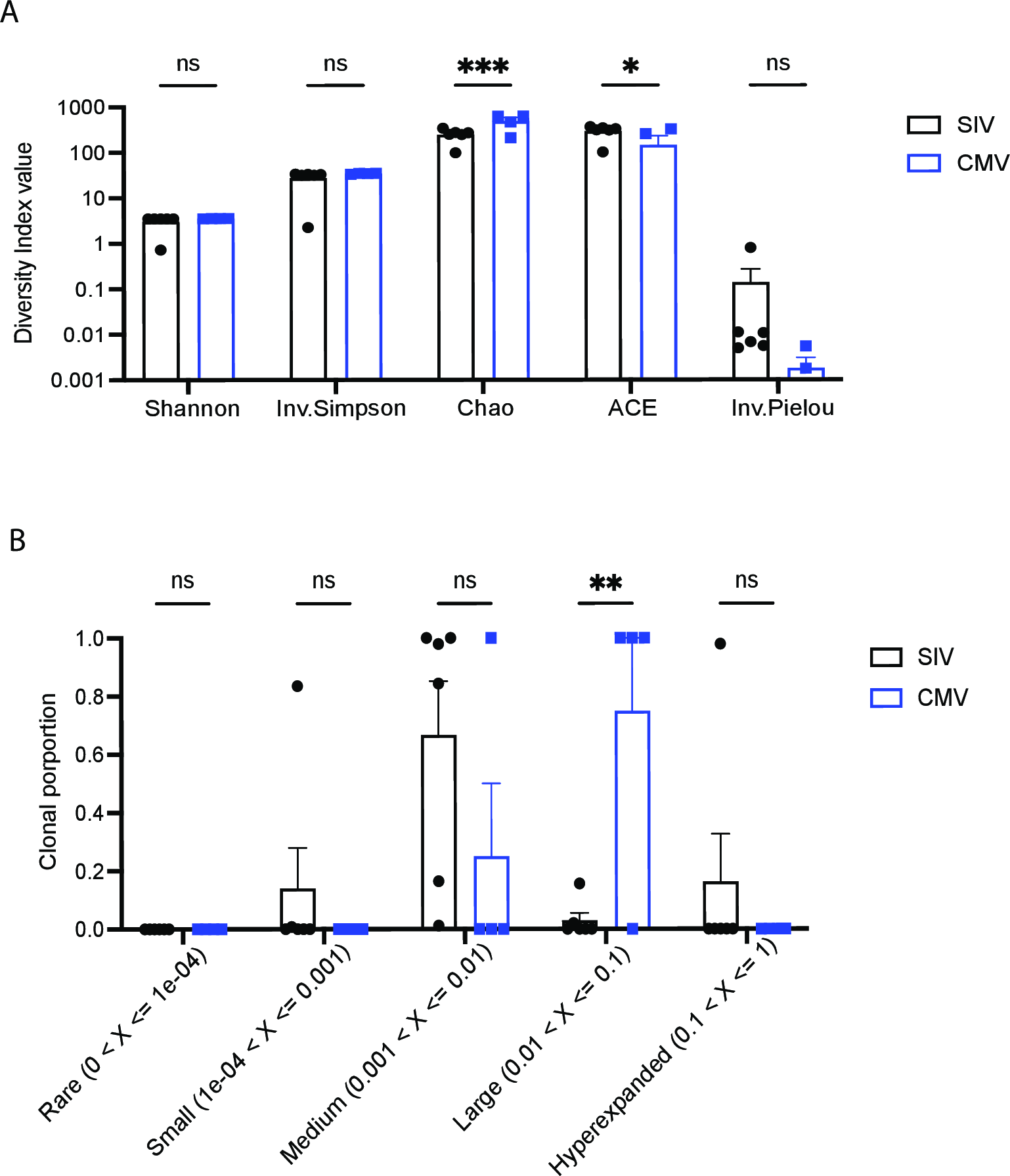

Supplement: S3 Fig — (A) Diversity index values for SIV CM9 and CMV VY9-specific CD8+ T cells in all anatomical sites. (B) Relative abundance of rare TCR sequences (0 < X < = 1e−04), small (low occurrence) TCR sequences (1e−04 < X < = 0.001), medium TCR sequences (0.001 < X < = 0.01), large TCR sequences (0.01 < X < = 0.1) and hyperexpanded TCR sequences (0.1 < X < = 1). Data are representative of 6 animals (CM9) and 4 animals (VY9). Data are represented as mean with SD. Statistical comparisons were achieved via 2-way ANOVAs with Sidak’s multiple comparisons test, with significance defined as p < 0.05. (TIF) [file ppat.1012545.s005.tif]

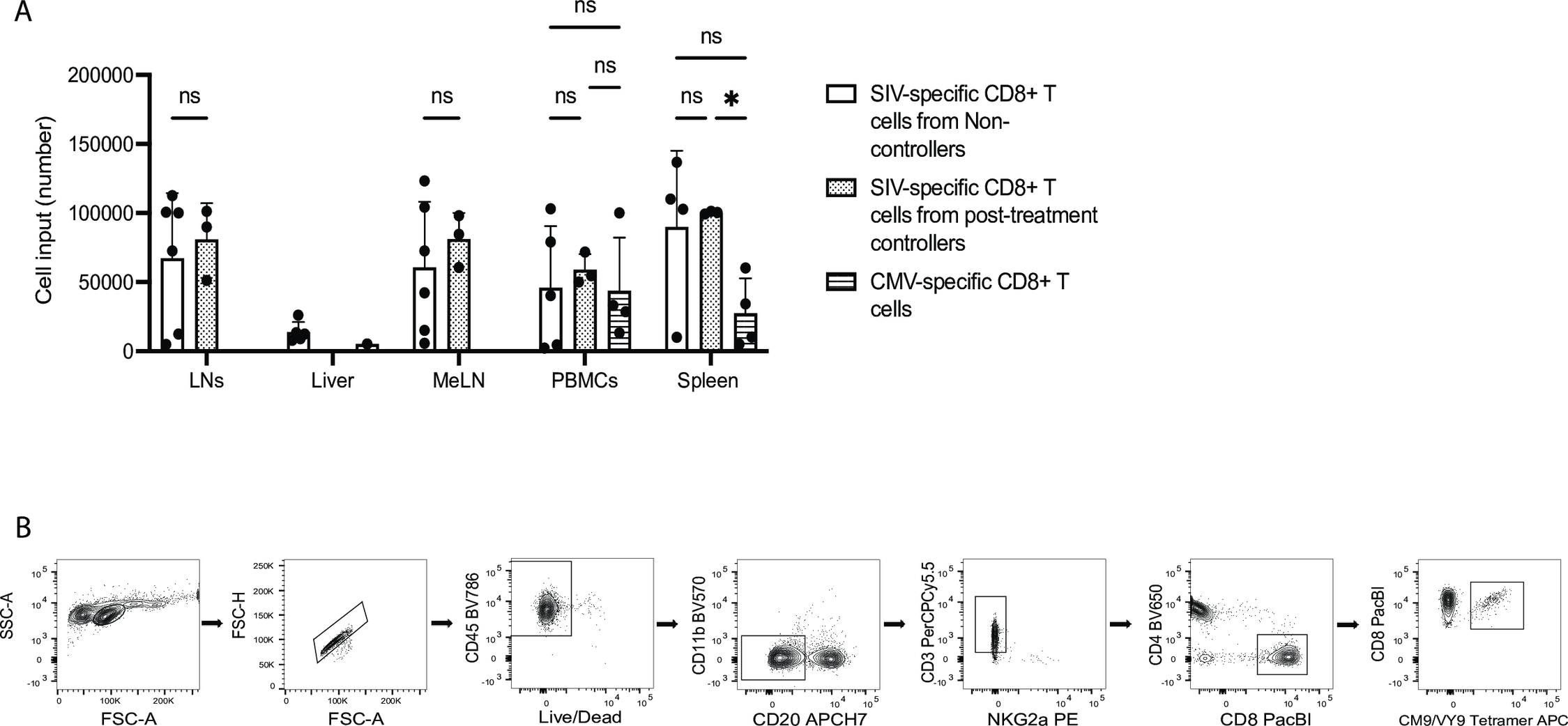

Supplement: S4 Fig — (A) Total number of sorted SIV CM9 or CMV VY9-specific CD8+ T cells from all animals in each study. (B) Gating strategy for sorting SIV CM9 or CMV VY9-specific CD8+ T cells in all anatomical sites. Data are represented as individual values and means (A) and flow plots (B). Statistical comparisons (A) were achieved via multiple effects analysis with Tukey’s multiple comparisons test, with significance defined as p < 0.05. (TIF) [file ppat.1012545.s006.tif]
